# Supplementary material for: A lipophilic cation protects crops against fungal pathogens by multiple modes of action
Source: Nat Commun. 2020 Mar 30;11:1608. doi: 10.1038/s41467-020-14949-y (PMC7105494; doi:10.1038/s41467-020-14949-y)
Supplement: Supplementary file 1 — Supplementary Information [file 41467_2020_14949_MOESM1_ESM.pdf]

A lipophilic cation protects crops against fungal pathogens by multiple modes of action

(Steinberg et al.)

Contents

Supplementary Figures

Supplementary Figure 1 - The “branched” mitochondrial respiration chain in fungi is a potential target for lipophilic cations.
Supplementary Figure 2 - Ultrastructure of C_{12}-G^{+}-induced plasma membrane invaginations in *Z. tritici*.
Supplementary Figure 3 - The effect of high concentrations of C_{12}-G^{+} on mitochondrial morphology and membrane potential in human C109 cells.
Supplementary Figure 4 - Mitochondrial fragmentation and inhibition of respiration in *Z. tritici* cells, treated with various MALCs and related chemistries.
Supplementary Figure 5 - Ultrastructure of *Z. tritici* mitochondria, treated for 30 minutes with various compounds.
Supplementary Figure 6 - Sulfonium lipophilic cations used in this study.
Supplementary Figure 7 - The effect of MALCs on the rice blast fungus *M. oryzae* and the corn smut fungus *U. maydis*.
Supplementary Figure 8 - Phytotoxicity of MALCs and plant disease symptoms.
Supplementary Figure 9 – MTT testing of human HepG2 cells.

Supplementary Tables

Supplementary Table 1 – Molecule SMILES notations and estimated LogP values.
Supplementary Table 2 - Strains and plasmids used in this study.
Supplementary Table 3 - Experimental usage of strains.
Supplementary Table 4 - AMES testing of C_{18}-SMe_2^{+}, no metabolic activation (-S9).
Supplementary Table 5 - AMES testing of C_{18}-SMe_2^{+} with metabolic activation (+S9).
Supplementary Table 6 - Comparison of efficacy of C_{18}-SMe_2^{+} with C_{12}-G^{+} (dodine).
Supplementary Table 7 - Experimental conditions for all data shown in this study.

Supplementary References

Supplementary Figures

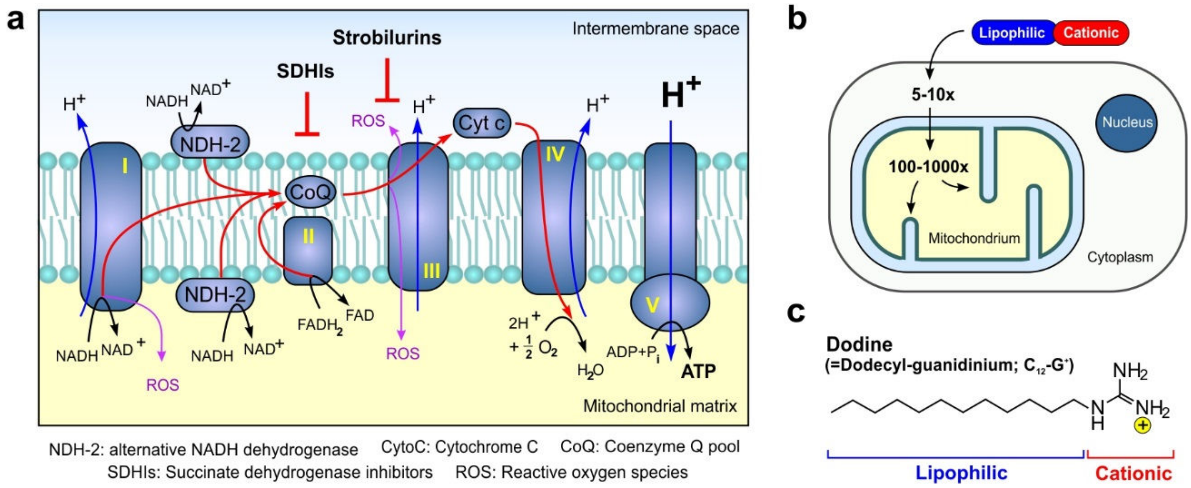

Supplementary Figure 1 The “branched” mitochondrial respiration chain in fungi is a potential target for lipophilic cations.

a Oxidative phosphorylation involves step-by-step electron transfer (red arrows) between 4 protein complexes of the mitochondrial respiration chain (numbered I-IV). In fungi, alternative membrane-associated NADH dehydrogenases participate in the respiration chain (NDH-2; ref. 1,2). Electron transfer is used to maintain a proton gradient over the inner membrane that is used to synthesise ATP[3]. In addition, healthy mitochondria produce low amounts of ROS, which serve various physiological functions in the cell[4].

b Lipophilic cations can cross the plasma membrane and accumulate in the cytoplasm and predominantly in the mitochondrial matrix (concentration factors are indicated). From here, they can insert into the inner mitochondrial membrane, where they could interfere with the fine-tuned interplay between the complexes of the respiration chain. Concentration factors were taken from references[5,6].

c C_{12}-G^+, the active ingredient of the fungicide Syllit (former name dodine), is a mono-alkyl lipophilic cation (MALC).

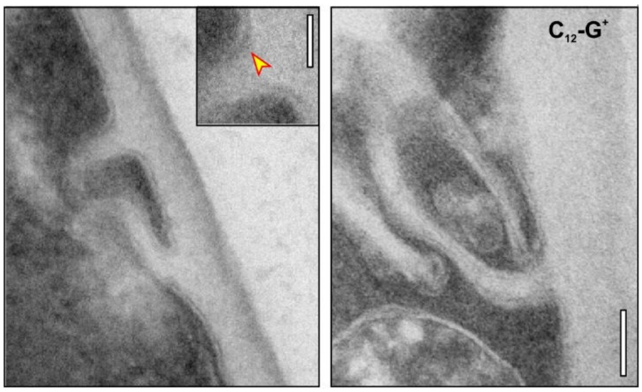

Supplementary Figure 2 Ultrastructure of C_{12}-G^+-induced plasma membrane invaginations in *Z. tritici*. Note that invaginations occur only at high concentrations. Scale bars=50 nm (insert in left panel) and 200 nm. See Supplementary Table 7 for experimental conditions. All source data are provided as a Source Data file.

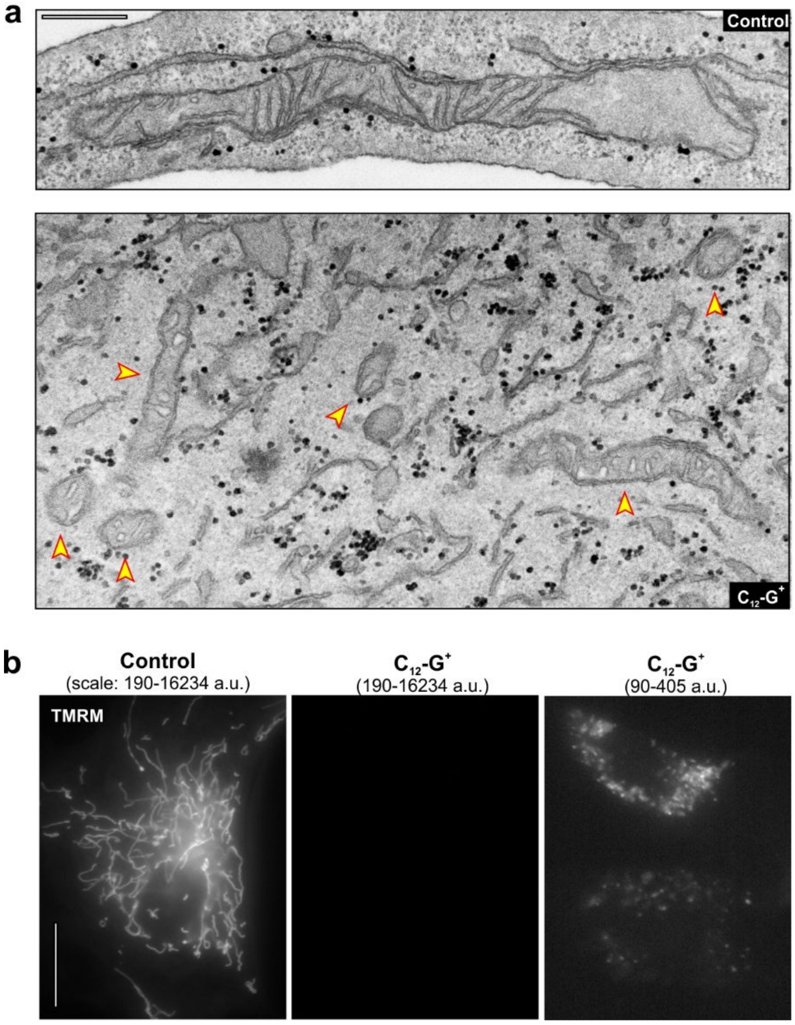

Supplementary Figure 3 The effect of C_{12}-G^+ on mitochondrial morphology and membrane potential in human skin fibroblasts.

a Electron micrograph of C_{12}-G^+-treated human skin fibroblast. Arrowheads indicate fragmented mitochondria. Note the C_{12}-G^+-induced alteration in cristae organization. Scale bar=250 nm.

b Methanol- and C_{12}-G^+-treated human skin fibroblasts, stained with the mitochondrial membrane potential reporter TMRM. Note that the middle and the right panel show the same 2 cells, but at different image scaling. Scaling range is provided in brackets. Scale bar=20 μm.

See Supplementary Table 7 for experimental conditions. All source data are provided as a Source Data file.

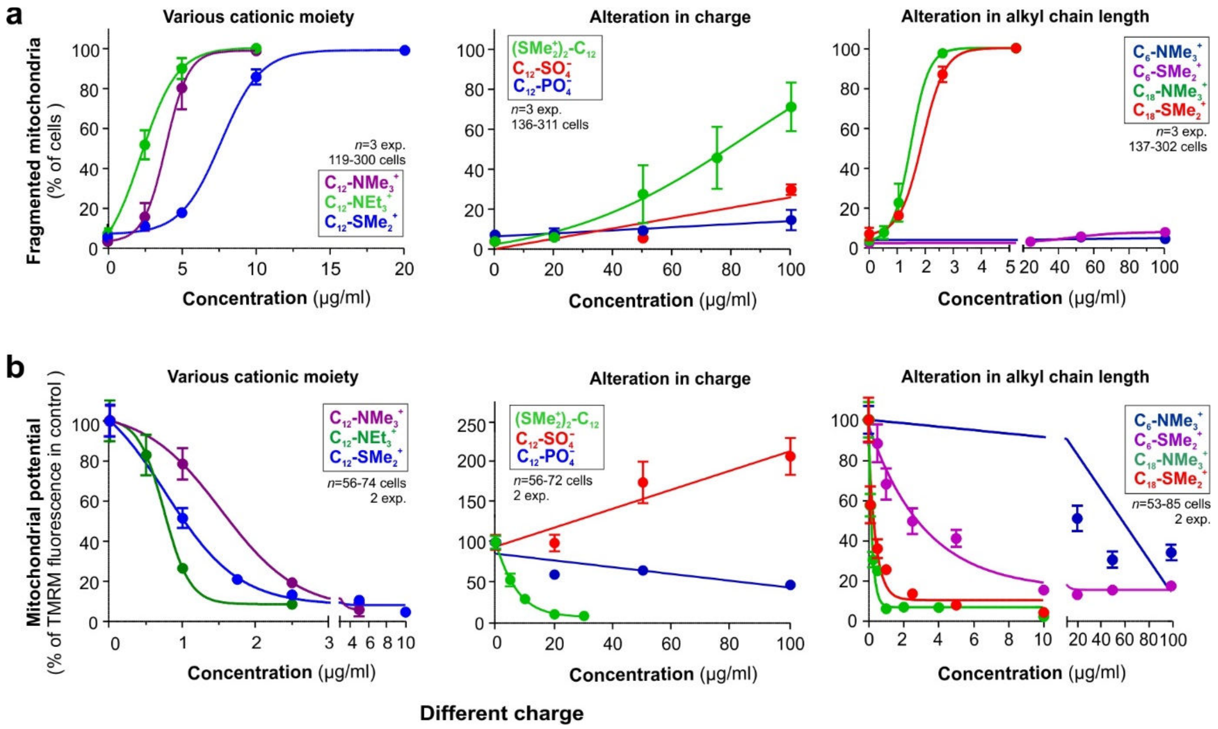

Supplementary Figure 4 Mitochondrial fragmentation (**a**) and depolarisation of mitochondria (**b**) in *Z. tritici* cells, treated with various MALCs and related chemistries. Values are given as mean ± SEM, sample sizes are indicated in graphs. Non-linear regression curves were calculated as dose-response inhibition (4 parameters) in

Prism5. See Supplementary Table 7 for experimental conditions. All source data are provided as a Source Data file.

.

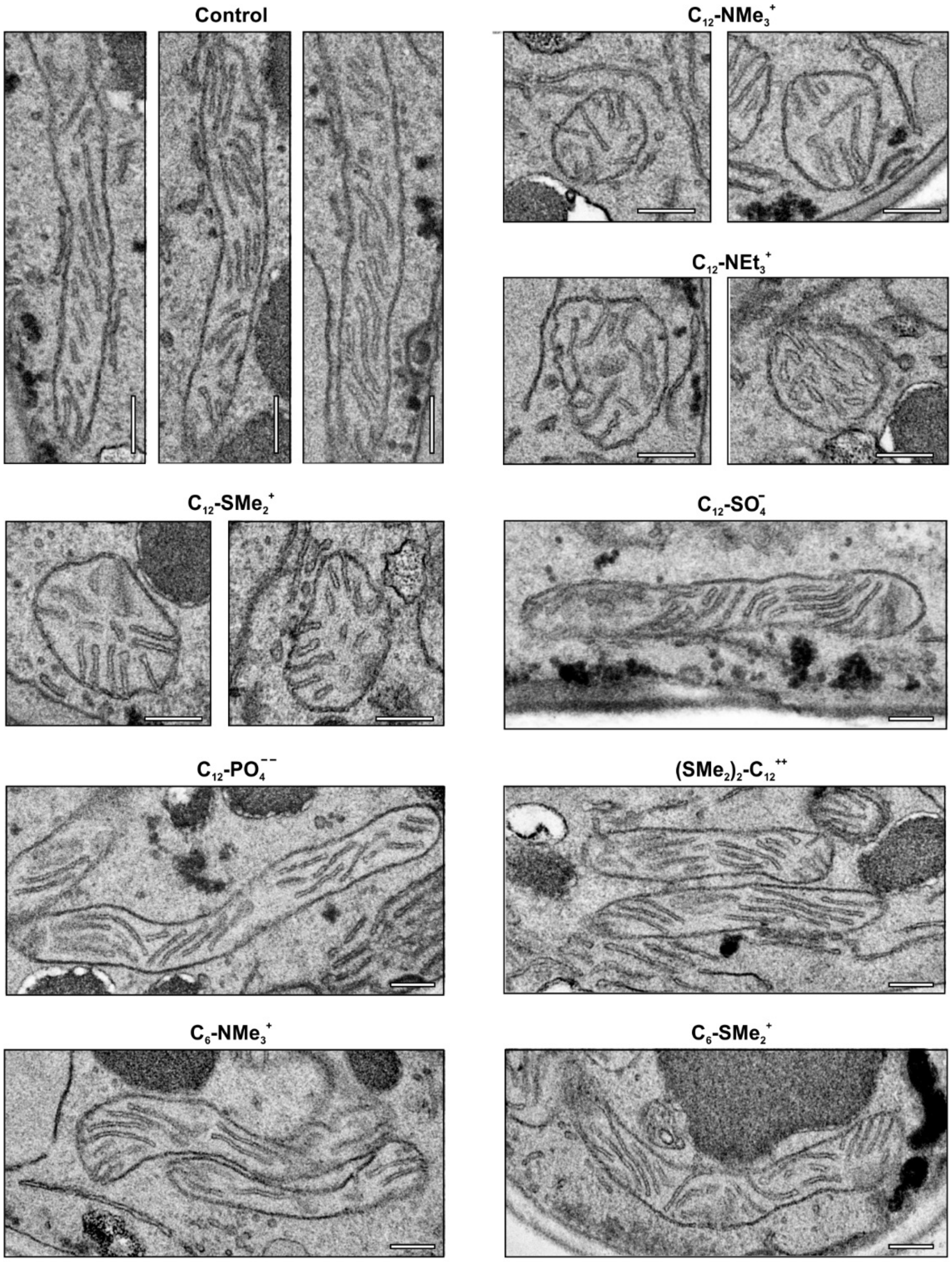

.

Supplementary Figure 5 Ultrastructure of *Z. tritici* mitochondria, treated for 30 minutes at room temperature with various compounds, all at 5 µg ml^{-1}. All scale bars=300 nm. All source data are provided as a Source Data file.

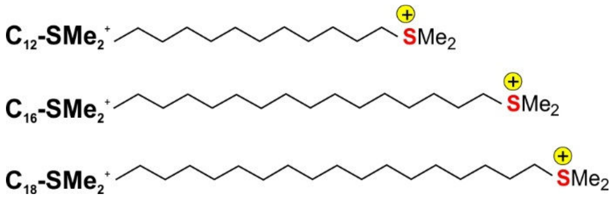

Supplementary Figure 6 Sulfonium cations used in this study. Note that only C_{18}-SMe_2^+ induces mROS production in *Z. tritici*.

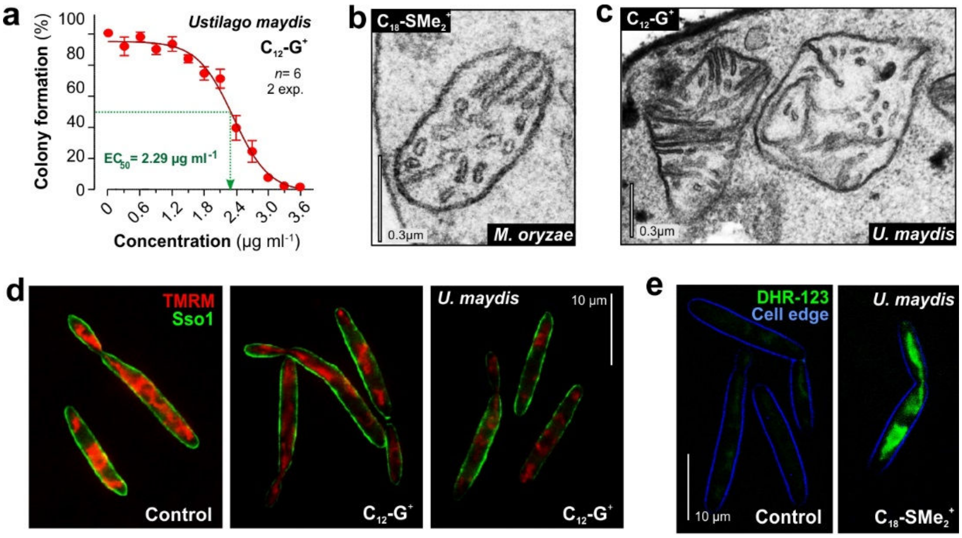

Supplementary Figure 7 The effect of MALCs on the corn smut fungus *U. maydis*.

a Colony formation in *U. maydis*, strain FB1, supplemented with increasing amounts of dodine. Values are mean ± standard error of the mean, sample size is n= 6. These data and the mean, 25th/75th percentiles, minimum/maximum, values Shapiro-Wilk P-values are provided in the Data Source file. Non-linear regression curve in was calculated as dose-response inhibition (4 parameters) in Prism5.

b Electron micrograph of a mitochondrion in a C_{18}-SMe_2^+-treated cell of *M. oryzae*. Scale bar=300 nm.

c Electron micrograph of mitochondria in a C_{12}-G^+-treated cell of *U. maydis.* Scale bar=300 nm.

d The mitochondrial membrane potential, visualised with TMRM, in *U. maydis*. The plasma membrane is labelled with GFP-Sso1 (green). Note that the TMRM image was image processed identically to allow comparison. Scale bar=10 µm.

e DHR-123 staining of mROS in *U. maydis* cells, treated with the solvent (Control) and C_{12}-G^+. The cell edge is given in blue. Scale bar=10 µm.

Values in (**a**) are given as mean ± SEM, sample sizes are n= 6. Non-linear regression curves were calculated as dose-response inhibition (4 parameters) in Prism5. See Supplementary Table 7 for experimental conditions. All source data are provided as a Source Data file.

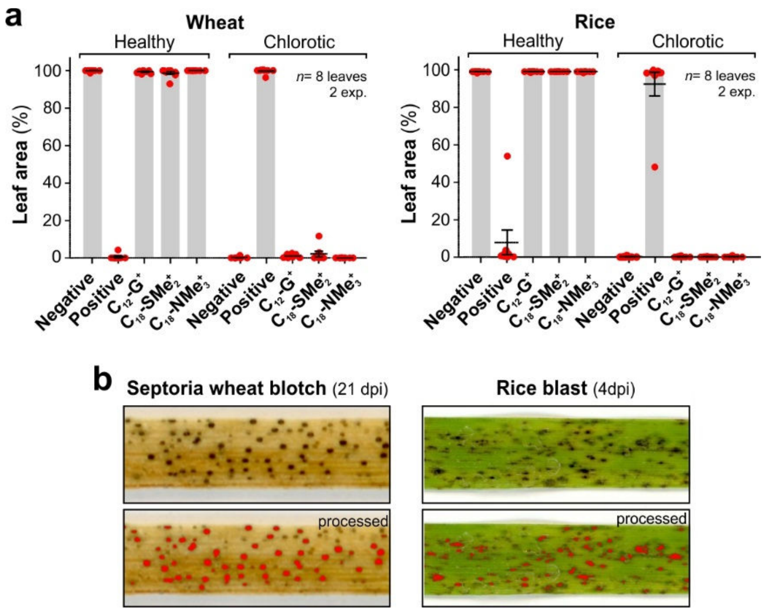

Supplementary Figure 8 Phytotoxicity of MALCs and plant disease symptoms.

a Chlorotic leaf regions in wheat and rice, sprayed with C_{12}-G^+, C_{18}-SMe_2^+ and C_{18}-NMe_3^+. Leaf discolouration was monitored at 21 days (wheat) and 4 days (rice) after infection.

b Symptoms of Septoria wheat blotch after 21 days and rice blast on leaves after 4 days. Lower panels show the automatic detection of dark pycnidia (Septoria wheat blotch) and necrotic lesions (rice blast), using the threshold function in the software package ImageJ (see Methods in main text).

Values represent the mean ± standard error of mean, with a sample size *n*= 8. Red dots indicate data points. See Supplementary Table 7 for experimental conditions. All source data are provided as a Source Data file.

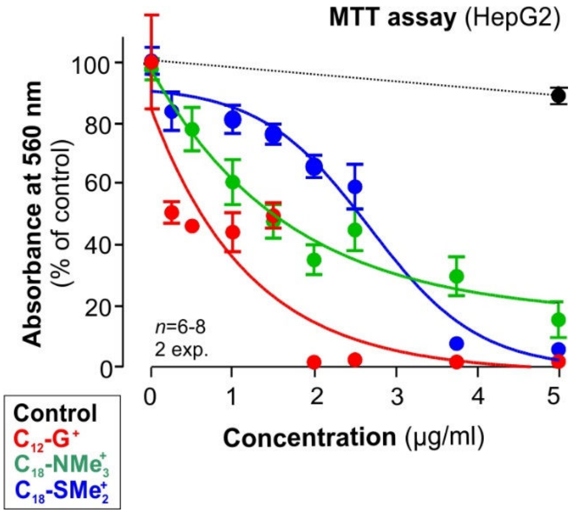

Supplementary Figure 9 MTT testing of cell proliferation and metabolic activity in human hepatocytes (HepG2).

MTT test for cell viability and metabolic activity in human HepG2 cells. See Table 1 in main text for EC_{50} values.

Values are given as mean ± SEM, sample sizes are n= 6-8. Non-linear regression curves were calculated as dose-response inhibition (4 parameters) in Prism5. See Supplementary Table 7 for experimental conditions. All source data are provided as a Source Data file.

Supplementary Tables

Supplementary Table1

Molecule SMILES notations and estimated LogP values

Molecule	SMILES	LogP
A) Molecules that do not diffuse through the membrane		
Glucose	OCC1OC(O)C(O)C(O)C1O	-2.26
ATP	NC1=NC=NC2=C1N=C[N]2C3OC(CO[P](O)(=O)O[P](O)(=O)O[P](O)(O)=O)C(O)C3O	-3.39
B) Molecules that stay in the membrane		
Dioleoylphosphatidic acid (phospholipid)	CCCCCCCC\C=C/CCCCCCCC(=O)OCC(CO[P](O)(O)=O)OC(=O)CCCCCCC\C=C/CCCCCCCC	10.19
C) Small molecule dyes that target mitochondria		
Rhodamine 123	COC(=O)C1=CC=CC=C1C2=C3C=CC(=[NH2+])C=C3OC4=C2C=CC(=C4)N	2.15
JC-1	CCN1C(=C\C=C\C2=[N+](CC)C3=CC(=C(Cl)C=C3[N]2CC)Cl)N(CC)C4=CC(=C(Cl)C=C14)Cl	5.02
TMRM	CCOC(=O)C1=C(C=CC=C1)C2=C3C=CC(C=C3OC4=C2C=CC(=C4)N(C)C)=[N+](C)C	2.74
D) Therapeutic molecules that target mitochondria		
MitoQ	O=C(C(OC)=C1OC)C(CCCCCCCCCC[P+](C2=CC=CC=C2)(C3=CC=CC=C3)C4=CC=CC=C4)=C(C)C1=O	7.41
Mito-Vitamin C	[O]C1=C(O)C(C(SCCCCCCCCCCC[P+](C2=CC=CC=C2)(C3=CC=CC=C3)C4=CC=CC=C4)O)OC1=O	5.40
Mito-Metformin	NC(NC(NCCCCCCCCCC[P+](C1=CC=CC=C1)(C2=CC=CC=C2)C3=CC=CC=C3)=N)=[NH2+]	3.94
E) Anti-fungal chemistries		
K20 (amphiphilic kanamycin)	NCC1OC(OC2C(O)C(OC3C(O)C(N)C(O)C(COS(=O)(CCCCCCCC)=O)O3)C(N)CC2N)C(O)C(O)C1O	-3.06
Azoxystrobin (targets mitochondria)	CO\C=C(\C(=O)OC)C1=C(OC2=NC=NC(=C2)OC3=CC=CC=C3C#N)C=CC=C1	2.97
Fluxapyroxat (targets mitochondria)	C[N]1C=C(C(=O)NC2=CC=CC=C2C3=CC(=C(F)C(=C3)F)F)C(=N1)C(F)F	4.33
F) Mono-alky lipophilic cations, analysed in this study		
C_{12}-G^+ (=dodine, Syllit)	CCCCCCCCCCCCNC(N)=[NH2+]	2.26
C_{18}-NMe_3^+	CCCCCCCCCCCCCCCCCC[N+](C)(C)C	4.24
C_{18}-SMe_2^+	CCCCCCCCCCCCCCCCCC[S+](C)C	5.96

Negative: low lipophilicity (extreme given in blue); Positive: high lipophilicity (extreme values in red). LogP values are averages from 5 predictions, done in SwissADME (http://www.swissadme.ch).

Supplementary Table 2 Fungal strains and plasmids used in this study

A. Strains	Genotype	Reference
Z. tritici		
IPO323	wildtype, MAT1-1	7
IPO323_eGFP-Sso1	*MAT1-1* / pCeGFPSso1	8
IP0323_Acd1-ZtGFP	*MAT1-1* / pCAcd1-ZtGFP	8
IPO323_mCh-ZtSso1	*MAT1-1* / pHmCherrySso1	This study
U. maydis		
FB1	wildtype*, a1 b1*	9
FB1GSso1	*a1 b1*/poGSso1	This study
M. oryzae		
Guy11	wildtype, MAT-2	10
B. Plasmids		
pCeGFPSso1	P*tub1-eGFP-sso1*, cbx^R	8
pCAcd1-ZtGFP	P*tub1-acd1-ZtGFP*, cbx^R	8
pHmCherrySso1	P*tub1-mCherry-sso1*, hyg^R	8
poGSso1	P*otef-egfp-sso*1, cbx^R	11

hyg^R or H, hygromycin resistance; cbx^R or C, carboxin resistance; *egfp*, enhanced green fluorescent protein; *Ztgfp*, *Z. tritici* codon optimised enhanced green fluorescent protein; *mCherry*, monomeric cherry; *sso*1, a syntaxin-like plasma membrane protein; *acd1*, putative acyl-CoA dehydrogenase; MAT, Mating type; P*otef*, constitutive promoter; P*tub1*, αtubulin promoter

Supplementary Table 3 Experimental usage of fungal strains

Strain name	Type of experiment	Figure reference
Z. tritici		
IPO323	Plate growth; plant protection assays; electron microscopy; ATP analysis; Complex 1 activity assay; O_2 consumption analysis	Fig. 1a, f; Fig. 2i, k; Fig. 4e, f; Fig 7c, e, g; Fig. S8b
IPO323_eGFP-Sso1	Live/Dead staining; membrane appearance; electron microscopy; mitochondrial potential measurement	Fig. 1b-e, g,h; Fig. 2e, f; Fig. 3d; Fig. 4d, h; Fig. 8b; Fig. S2; Fig. S4b
IP0323_Acd1-ZtGFP	Mitochondrial morphology; electron microscopy; mitochondria functionality after purification	Fig. 2a, d; Fig 2j; Fig. 3b; Fig. 4 b, c; Fig. S4a; Fig. S5
IPO323_mCh-ZtSso1	plasma membrane permeability; mROS and Apoptosis analysis	Fig. 1i-k; Fig. 4g; Fig. 5a-h; Fig. 8a
U. maydis		
FB1	Plate growth; mROS analysis	Fig. 6f; Fig. S7a, c, e
FB1GSso1	Mitochondrial potential analysis	Fig. 6d, Fig. S7d
M. oryzae		
Guy11	Plant protection; effect of C_{12}-G^+, C_{18}-NMe_3^+ and C_{18}-SMe_2^+on germination and mitochondria; electron microscopy	Fig. 6a, b, c, e; Fig. 7d, f, h; Fig. S7b; Fig. S8b

Supplementary Table 4

Ames testing of C_{18}-SMe_2^+ without metabolic activation (-S9)

(taken from a study report, provided by Gentronix Ltd., Macclesfield, UK)

Study number: AME00744
Start date: 25 June 2019
Test item: C18-DMS/C18-SMe2+
Solvent: Dimethyl sulphoxide

Experiment: AME00744(01)
End date: 28 June 2019
Batch: Not provided

(Dose per plate)		Number of revertant colonies per plate [mean number of revertant colonies per plate]									
		S. typhimurium LT2								*E. coli* WP2	
µg	µmol	TA1535		TA1537		TA98		TA100		*uvrA* /pKM101	
Solvent control		12	12	9	9	27	29	127	130	146	150
		13	*0.6*	9	*0.6*	31	*2.0*	134	*3.6*	151	*4.0*
		12		8		29		129		154	
0.05	0.000216	14	14	10	9	31	29	143	134	148	146
		13	*0.6*	8	*1.0*	29	*2.0*	130	*8.1*	149	*4.4*
		14	**1.2**	9	**1.0**	27	**1.0**	128	**1.0**	141	**1.0**
0.16	0.000691	14	14	8	9	26	26	127	129	153	149
		16	*1.5*	8	*1.2*	25	*1.0*	128	*2.6*	147	*3.8*
		13	**1.2**	10	**1.0**	27	**0.9**	132	**1.0**	146	**1.0**
0.5	0.00216	12	12	10	8	29	27	129	127	151	159
		11	*1.0*	7	*1.5*	26	*1.5*	127	*1.5*	153	*12.7*
		13	**1.0**	8	**0.9**	27	**0.9**	126	**1.0**	174	**1.1**
1.6	0.00691	10	10	9	9	22	20	120	123	131	140
		9	*1.0*	10	*1.0*	18	*2.0*	127	*3.8*	142	*8.2*
		11	**0.8**	8	**1.0**	20	**0.7**	121	**0.9**	147	**0.9**
5	0.0216	11	10	10	10	23	21	118	119	149	149
		8	*1.5*	11	*1.5*	16	*4.7*	117	*3.2*	156	*7.0*
		10	**0.8**	8	**1.1**	25	**0.7**	123	**0.9**	142	**1.0**
16	0.0691	11 r	11	9 r	9	29 r	24	102 r	104	139 s	143
		11 r	*0.6*	8 r	*0.6*	23 r	*4.2*	101 r	*3.8*	146 s	*3.6*
		10 r	**0.9**	9 r	**1.0**	21 r	**0.8**	108 r	**0.8**	144 s	**1.0**
35	0.151	6 v	6	5 v	5	11 v	11	55 v	51	122 r	125
		7 v	*1.5*	4 v	*1.0*	12 v	*1.0*	40 v	*9.6*	127 r	*2.5*
		4 v	**0.5**	6 v	**0.6**	10 v	**0.4**	58 v	**0.4**	125 r	**0.8**
50	0.216	0 f	0	0 f	0	0 f	0	0 f	0	92 v	92
		0 f	*0.0*	0 f	*0.0*	0 f	*0.0*	0 f	*0.0*	91 v	*1.5*
		0 f	**0.0**	0 f	**0.0**	0 f	**0.0**	0 f	**0.0**	94 v	**0.6**
Positive control		473	460	208	232	309	299	488	528	847	901
		449	*12.2*	262	*27.4*	292	*9.1*	536	*36.2*	957	*55.0*
		457	**38.3**	227	**25.8**	295	**10.3**	559	**4.1**	899	**6.0**

Positive controls

TA1535:	sodium azide	0.5 µg/plate
TA1537:	9-aminoacridine.HCl	50 µg/plate
TA98:	2-nitrofluorene	1 µg/plate
TA100:	sodium azide	0.5 µg/plate
uvrA /pKM101:	potassium dichromate	25 µg/plate

Comments

s: slightly reduced lawn
r: reduced lawn
v: very reduced lawn
f: pseudorevertants

[box] = mean   *italics* = Standard Deviation   **bold** = fold increase over solvent control

TA1535 and TA1537 solvent control and test item plates were scored manually, all other plates were scored using Sorcerer image analysis system.

Supplementary Table 5

Ames testing of C_{18}-SMe_2^+ with metabolic activation (+S9)

(taken from a study report, provided by Gentronix Ltd., Macclesfield, UK)

Study number: AME00744
Start date: 09 July 2019
Test item: C18-DMS/C18-SMe2+
Solvent: Dimethyl sulphoxide

Experiment: AME00744(03)
End date: 12 July 2019
Batch: Not provided

(Dose per plate)		Number of revertant colonies per plate [mean number of revertant colonies per plate]									
		S. typhimurium LT2								*E. coli* WP2	
µg	µmol	TA1535		TA1537		TA98		TA100		*uvrA* /pKM101	
Solvent control		12	11	12	12	32	36	142	150	196	197
		10	*1.2*	12	*0.6*	34	*5.3*	156	*7.1*	200	*3.1*
		12		13		42		151		194	
0.5	0.00216	15	14	10	11	30	34	147	153	190	203
		12	*2.1*	11	*1.5*	30	*7.5*	155	*4.9*	212	*11.5*
		16	**1.3**	13	**0.9**	43	**0.9**	156	**1.0**	207	**1.0**
1.6	0.00691	13	13	11	11	35	37	164	152	201	215
		15	*2.5*	10	*0.6*	39	*2.1*	155	*14.3*	223	*12.4*
		10	**1.2**	11	**0.9**	38	**1.0**	136	**1.0**	222	**1.1**
5	0.0216	10	9	13	11	32	31	159	163	182	202
		9	*0.6*	10	*1.7*	29	*1.7*	146	*18.8*	193	*25.7*
		9	**0.8**	10	**0.9**	32	**0.9**	183	**1.1**	231	**1.0**
16	0.0691	9	9	12	13	30	30	157	161	221	219
		9	*0.6*	11	*2.1*	31	*1.0*	179	*15.9*	210	*8.2*
		8	**0.8**	15	**1.1**	29	**0.8**	148	**1.1**	226	**1.1**
50	0.216	11	12	13	15	32	32	146	150	182	212
		12	*1.0*	15	*1.5*	31	*0.6*	149	*4.6*	208	*31.7*
		13	**1.1**	16	**1.3**	32	**0.9**	155	**1.0**	245	**1.1**
160	0.691	10 rp	8	11 rp	12	30 rp	28	140 rp	135	160 p	159
		6 rp	*2.1*	13 rp	*1.2*	26 rp	*2.1*	143 rp	*10.8*	165 p	*7.1*
		7 rp	**0.7**	13 rp	**1.0**	29 rp	**0.8**	123 rp	**0.9**	151 p	**0.8**
500	2.16	0 fp	0	0 fp	0	0 fp	0	0 fp	0	53 rp	63
		0 fp	*0.0*	0 fp	*0.0*	0 fp	*0.0*	0 fp	*0.0*	60 rp	*11.2*
		0 fp	**0.0**	0 fp	**0.0**	0 fp	**0.0**	0 fp	**0.0**	75 rp	**0.3**
1600	6.91	0 np	0	0 np	0	0 np	0	0 np	0	0 fp	0
		0 np	*0.0*	0 np	*0.0*	0 np	*0.0*	0 np	*0.0*	0 fp	*0.0*
		0 np	**0.0**	0 np	**0.0**	0 np	**0.0**	0 np	**0.0**	0 fp	**0.0**
3500	15.1	0 np	0	0 np	0	0 np	0	0 np	0	0 np	0
		0 np	*0.0*	0 np	*0.0*	0 np	*0.0*	0 np	*0.0*	0 np	*0.0*
		0 np	**0.0**	0 np	**0.0**	0 np	**0.0**	0 np	**0.0**	0 np	**0.0**
5000	21.6	0 np	0	0 np	0	0 np	0	0 np	0	0 np	0
		0 np	*0.0*	0 np	*0.0*	0 np	*0.0*	0 np	*0.0*	0 np	*0.0*
		0 np	**0.0**	0 np	**0.0**	0 np	**0.0**	0 np	**0.0**	0 np	**0.0**
Positive control		165	149	133	158	1511	1521	1975	2021	2946	3026
		142	*13.9*	157	*25.5*	1525	*9.1*	1905	*145.2*	2970	*118.4*
		140	**13.5**	184	**13.2**	1528	**42.3**	2184	**13.5**	3162	**15.4**

Positive controls

TA1535: 2-aminoanthracene 2 µg/plate
TA1537: 2-aminoanthracene 2 µg/plate
TA98: 2-aminoanthracene 2 µg/plate
TA100: 2-aminoanthracene 2 µg/plate
uvrA /pKM101: 2-aminoanthracene 20 µg/plate

Comments

r: reduced lawn
f: pseudorevertants
n: no survival
p: precipitate

[ ] = mean *italics* = Standard Deviation **bold** = fold increase over solvent control

TA1535 and TA1537 solvent control and test item plates were scored manually, all other plates were scored using Sorcerer image analysis system.

Supplementary Table 6. Comparison of efficacy of C_{18}-SMe_2^+ with C_{12}-G^+ (dodine)

	Fold-change
1. Toxicity in *Z.tritici*	
Fragmentation of mitochondria	+3.30
Depolarisation of mitochondria	+1.31
Inhibition of ATP synthesis	+2.96
Cell mortality	+2.10
2. Toxicity in human cells	
Fragmentation of mitochondria	+1.35
Inhibition of respiration*	-1.44
MTT tested cytotoxicity (C109)	+1.52
MTT tested cytotoxicity (HepG2)	+3.71
3. Toxicity in zooplankton	
Mortality of *Daphnia magna*	+7.81
Relative toxicity	+16.40
4. Toxicity in plants	
Leaf symptoms in wheat	No toxicity found
Leaf symptoms in rice	No toxicity found
5. Anti-fungal protection	
Against Septoria leaf blotch	+22.7
Against rice blast disease	+6.60
Inhibition of *M. oryzae* germination	+1.09
In induction of plant defence	+4.50

All values indicate a fold-change in anti-fungal performance or toxicity of C_{18}-SMe_2^+ compared to C_{12}-G^+; values are based on data summarised in Table 1; Improved performance is indicated by "+"; decreased performance is indicated by "-".

Supplementary Table 7. Experimental conditions for all data shown in this study.

Fig. 1	(a) var. conc. (=various concentrations), 5 days, 18°C, (b) 100 µg ml^{-1}, 3 h, 18°C; (c) var. conc. and times, 18°C; (d) 100 µg ml^{-1}, 30 min, RT, (e) var. conc., 30 min, RT, (f) 100 µg ml^{-1}, 30 min, RT, (g) 50 µg $ml^{-1,}$ 30 min, RT; (h) var. conc., 30 min, RT, (i) 50 µg ml^{-1}, 30 min, RT; (j) 100 µg ml^{-1}, 30 min, RT; (k) var. conc., 30 min, RT; control= 0.5 % (v/v) methanol in media.
Fig. 2	(a) 10 µg ml^{-1}, 30 min, RT; (b) var. conc., 30 min, RT; (d) 10 µg ml^{-1}, 30 min, RT, (e) 0.5 and 1 µg ml^{-1}, 30 min, RT; (f) var. conc. , 30 min, RT; (g) 5 µg ml^{-1}, var. time, RT; (i) 5 µg ml^{-1}, 3h, RT; (k) 100 µM (Rotenone), 50 µM (DPI), 5 µg ml-1 (C_{12}-G^+), 60 min, RT; control= 0.1 % (v/v) methanol in media.
Fig. 3	(a) 50 µg ml^{-1}, 30 min, 37°C; (b) var. conc., 30 min, 37°C (human) or RT (*Z. tritici*) (c) 50 µg ml^{-1}, 30 min, 37°C; (d) var. conc., 30 min, 37°C (human) or RT (*Z. tritici*); control= 0.1 % (v/v) methanol in media.
Fig. 4	(b) 2.5 µg ml^{-1}, 30 min, RT; (c) 5 µg ml^{-1}, 30 min, RT; (d) 0.25 µg ml^{-1}, 30 min, RT; (e) 5 µg ml^{-1}, 120 min, RT; (f) 5 µg ml^{-1}; 60 min, RT; (g) 17 µg ml^{-1} (C_{12}-G^+), 14 µg ml^{-1} (C_{18}-SMe_2^+), 7 µg ml^{-1} (C_{18}-NMe_3^+), 30 min, RT; (h) 10 µg ml^{-1},24 h, RT; control= 0.1 % (v/v) methanol in media.
Fig. 5	(a) 5 µg ml^{-1} (C_{12}-G^+, C_{18}-NMe_3^+) and 100 µM (rotenone), 30 min, RT; (b) 5 µg ml^{-1}, 30 min, RT; (c) 5 µg ml^{-1} (C_{18}-SMe_2^+) and 100 µM (rotenone), 30 min, RT; (d) 5 or 20 µg ml^{-1} (as indicated), 30 min, RT; (e) 5 µg ml^{-1}, 24 h, RT; (f) 5 µg ml^{-1},24 h, RT; (g) 5 µg ml^{-1}, 24 h, RT; (h) 5 µg ml^{-1}, 24h, RT; ; all experiments were performed at room temperature; control= 0.1 % (v/v) methanol in media.
Fig. 6	(a) 10 µg ml^{-1} (C_{12}-G^+), 7.5 µg ml^{-1} (C_{18}-SMe_2^+), 5 µg ml^{-1} (C_{18}-NMe_3^+), 3h, 25°C; (b) var, conc., 3h, 25°C (c) 1 µg ml^{-1}, 30 min, RT; (d) 5 µg ml^{-1}, 30 min, RT; control= 0.1 % (v/v) methanol in media.
Fig. 7	(a) 1000 µg ml^{-1}, 7 d, 20-25ºC; (b) 1000 µg ml^{-1}, 7 d, 23-35ºC; (c) 100 µg ml^{-1}, 24 h, followed by infection with $3x10^5$ spores ml^{-1} (strain IPO323) and 21 d, 20-25ºC; (d) 125 µg ml^{-1}, 24 h, followed by infection with 1 x 10^5 spores ml^{-1} (strain Guy11), 4 d, 23-35ºC; (e) var. conc., 24h, followed by infection and 21 d, 20-25ºC; (f) var. conc., 24 h, followed by infection and 4 d, 23-35ºC; (g) 100 µg ml^{-1}, 24h, followed by infection, 21 d, 20-25ºC; (h) 125 µg ml^{-1}, 24 h, followed by infection, 4d, 23-35ºC; (i) 100 µg ml^{-1}, 6 h, 23-35ºC; (j) 100 µg ml^{-1}, 6 h, 23-35ºC; (k) 14 d-old rice plants sprayed with 150 µg ml^{-1} C_{18}-SMe_2^+, followed by an identical treatment after additional 14 d, 23-35ºC; (l) 14 d-old plants sprayed with 100 or 150 µg ml^{-1} C_{18}-SMe_2^+, followed by an identical treatment after additional 14 d, 23-35ºC; Controls are: (a) 0.4 % (v/v) methanol (Negative), 10 % (v/v) Tween20 (Positive), (b) 0.625 % (v/v) methanol (Negative) and 10 % (v/v) Tween20 (Positive), (c) 0.4 % (v/v) methanol/ 0.04 % (v/v) Tween20 (Negative) and $3x10^5$ spores ml^{-1} (strain IPO323, Positive), (d) 0.625% (v/v) methanol and 0.2 % (v/v) gelatine (Negative), 1 x 10^5 spores ml^{-1} (strain Guy11, Positive), (e) 0.4 % (v/v) methanol and 0.04 % (v/v) Tween20, (f) 0.625 % (v/v) methanol and 0.2 % (v/v) gelatine, (i, j) 0.4 % (v/v) methanol and 0.04 % (v/v) Tween20 (Negative), 15 mM salicylic acid (Positive).
Fig. 8	(a, b) var. conc., 30 min, RT (*Z. tritici*) or 37ºC (human); (c) var. conc., 24h, 37ºC ; (d) 1 µg ml^{-1}, 30 min, RT; (e) var. conc. 24 h, RT; (f) 0.1 (left panel) and 10 µg ml^{-1} (right panel) of C_{18}-SMe_2^+, (f, g) 1 µg ml^{-1} 2-nitrofluorene (Positive), 100 µl each plate dimethylsulfoxide (Negative), 0.1 µg ml^{-1}, 24h, 37ºC (f, C_{18}-SMe_2^+) and var. conc. of C_{18}-SMe_2^+ 24h, 37ºC;
Fig. S2	100 µg ml^{-1}, 30 min, RT
Fig. S3	(a, b) 35 µg ml^{-1}, 30 min, 37ºC
Fig. S4	(a, b) Var. conc., 30 min, RT
Fig. S5	5 µg ml^{-1}, 30 min, RT
Fig. S7	(a) var. conc., 5 d, 28ºC; (b) 10 µg ml^{-1}, 30 min, RT; (c) 5 µg ml^{-1}, 30 min, RT; (d) 3 µg ml^{-1} (middle panel), 5 µg ml^{-1} (right panel), 30 min, RT; (e) 1 µg ml^{-1}, 30 min, RT;
Fig. S8	(a) 1000 µg ml^{-1}, 7 d, 20-25ºC (wheat) and 1000 µg ml^{-1}, 7 d, 23-35ºC (rice); controls are: (a) 0.4 % (v/v) methanol (Negative), 10% (v/v) Tween 20 (Positive); (b) 0.625 % (v/v) methanol (Negative) and 10 % (v/v) Tween 20 (Positive).
Fig. S9	Var. conc., 24h, 37ºC

Supplementary References

1 Affourtit, C., Heaney, S.P. & Moore, A.L. Mitochondrial electron transfer in the wheat pathogenic fungus *Septoria tritici*: on the role of alternative respiratory enzymes in fungicide resistance. *Biochim. Biophys. Acta* **1459**, 291-298 (2000).

2 Joseph-Horne, T., Hollomon, D.W. & Wood, P.M. Fungal respiration: a fusion of standard and alternative components. *Biochim. Biophys. Acta* **1504**, 179-195 (2001).

3 Mitchell, P. Coupling of phosphorylation to electron and hydrogen transfer by a chemi-osmotic type of mechanism. *Nature* **191**, 144-148 (1961).

4 Thannickal, V.J. & Fanburg, B.L. Reactive oxygen species in cell signaling. *Am J Physiol. Lung Cell. Mol. Physiol.* **279**, L1005-1028 (2000).

5 Murphy, M.P. Targeting lipophilic cations to mitochondria. *Biochim. Biophys. Acta* **1777**, 1028-1031 (2008).

6 Zielonka, J. *et al.* Mitochondria-targeted triphenylphosphonium-based compounds: syntheses, mechanisms of action, and therapeutic and diagnostic applications. *Chem. Rev.* **117**, 10043-10120 (2017).

7 Kema, G.H.J. & van Silfhout, C.H. Genetic variation for virulence and resistance in the wheat-Mycosphaerella graminicola pathosystem. III. Comparative seedling and adult plant experiments. *Phytopathol.* **87**, 266–272 (1997).

8 Kilaru, S., Schuster, M., Ma, W. & Steinberg, G. Fluorescent markers of various organelles in the wheat pathogen *Zymoseptoria tritici*. *Fungal Genet. Biol.* **105**, 16-27 (2017).

9 Banuett, F. & Herskowitz, I. Different a alleles of *Ustilago maydis* are necessary for maintenance of filamentous growth but not for meiosis. *Proc. Natl. Acad.Sci. U S A* **86**, 5878-5882 (1989).

10 Leung, H., Borromeo, E., Bernardo, M. & Notteghem, J.L. Genetic analysis of virulence in the rice blast fungus *Magnaporthe grisea*. *Phytopathol.* **78**, 1227-1233 (1988).

11 Steinberg, G. & Schuster, M. The dynamic fungal cell. *Fungal Biol. Rev.* **25**, 14-37 (2011).
