# Supplementary material for: A lipophilic cation protects crops against fungal pathogens by multiple modes of action
Source: Nat Commun. 2020 Mar 30;11:1608. doi: 10.1038/s41467-020-14949-y (PMC7105494; doi:10.1038/s41467-020-14949-y)
Supplement: Supplementary file 3 — Description of Additional Supplementary Files [file 41467_2020_14949_MOESM3_ESM.pdf]

Description of Additional Supplementary Files

File Name: Supplementary Movie 1

Description: *Daphnia magna* after 24 h incubation at room temperature with 1 µg/ml C_{12}-G^+, C_{18}-NMe_3^+ and C_{18}-SMe_2^+.
